# From the Discovery of Extremozymes to an Enzymatic Product: Roadmap Based on Their Applications

**DOI:** 10.3389/fbioe.2021.752281

**Published:** 2022-01-12

**Authors:** Giannina Espina, Sebastián A. Muñoz-Ibacache, Paulina Cáceres-Moreno, Maximiliano J. Amenabar, Jenny M. Blamey

**Affiliations:** ^1^ Fundación Biociencia, Santiago, Chile; ^2^ Facultad de Química y Biología, Universidad de Santiago de Chile, Santiago, Chile

**Keywords:** biocatalysts, extremophiles, catalase, laccase, amine-transaminase, Antarctica

## Abstract

With the advent of the industrial revolution, the use of toxic compounds has grown exponentially, leading to a considerable pollution of the environment. Consequently, the development of more environmentally conscious technologies is an urgent need. Industrial biocatalysis appears as one potential solution, where a higher demand for more robust enzymes aims to replace toxic chemical catalysts. To date, most of the commercially available enzymes are of mesophilic origin, displaying optimal activity in narrow ranges of temperature and pH (i.e., between 20°C and 45°C, neutral pH), limiting their actual application under industrial reaction settings, where they usually underperform, requiring larger quantities to compensate loss of activity. In order to obtain novel biocatalysts better suited for industrial conditions, an efficient solution is to take advantage of nature by searching and discovering enzymes from extremophiles. These microorganisms and their macromolecules have already adapted to thrive in environments that present extreme physicochemical conditions. Hence, extremophilic enzymes stand out for showing higher activity, stability, and robustness than their mesophilic counterparts, being able to carry out reactions at nonstandard conditions. In this brief research report we describe three examples to illustrate a stepwise strategy for the development and production of commercial extremozymes, including a catalase from an Antarctic psychrotolerant microorganism, a laccase from a thermoalkaliphilic bacterium isolated from a hot spring and an amine-transaminase from a thermophilic bacterium isolated from a geothermal site in Antarctica. We will also explore some of their interesting biotechnological applications and comparisons with commercial enzymes.

## Introduction

The development of the biotechnology industry and the interest for greener and environmentally friendly technologies has led to an increasing demand for more efficient and less contaminant catalysts. Consequently, the global enzymes market has grown from US$2 billion in 2004 (Joseph et al., 2008), to $8.63 billion in 2019, and is expected to reach $14.5 billion by 2027 (Manjrekar and Wadekar, 2021). However, one of the main problems of using biocatalysts in industrial applications are the harsh conditions at which industrial processes usually take place. Under these settings, mesophilic enzymes that are currently the great majority of commercially available biocatalysts, perform poorly and require larger quantities to compensate for the loss of activity. Therefore, there is a great interest for more stable and efficient biocatalysts, better suited to work optimally at these extreme conditions (Atalah et al., 2019). This can be addressed *via* protein engineering of mesophilic enzymes, combining rational and computational design with directed evolution, to achieve the selectivity, catalytic efficiency and stability required for their industrial use, or by taking advantage of what is already available in Nature.

Extremophilic microorganisms and their macromolecules have already adapted to thrive in environments that present extreme physicochemical conditions. Extremozymes have adapted to optimally perform at, or close to the environmental conditions where extremophilic microorganisms inhabit (e.g., high or low temperatures, acidic or alkaline pH, high pressure, salinity, radiation) (Brininger et al., 2018). These novel extreme biocatalysts with increased stability to denaturing conditions, stand out for being highly efficient, showing higher activity, and robustness than their mesophilic counterparts, being able to carry out reactions at nonstandard conditions, producing low amounts of by-products under a wide range of extreme industrial settings (Gomes and Steiner, 2004; Cáceres-Moreno et al., 2019).

To date, there are two main approaches to discover novel extremozymes from environmental samples. On one hand, due to the inherent difficulty and specific requirements of *in vitro* culturing of extremophilic microorganisms, especially archaea, there is a culture independent approach, which is based on metagenomic sequencing followed by data mining to search for specific genes that potentially codify for an enzyme of interest (Sysoev et al., 2021). However, one disadvantage of this strategy is that to date, there is a lack of reliable functional annotation of extremophile genomic data, caused by the low amount of experimentally described genes. This translates in a shortfall of specific databases, with many genes getting annotated as hypothetical proteins with unknown functions, contributing to the problem of information shortage.

On the other hand, there is a culture dependent functional approach that allows to obtain native extremozymes able to catalyze reactions of interest at the desired conditions. For this, extremophiles are grown and isolated under the required selective pressures and then, enzymatic activity-based screening are performed to select the most promising ones for further studies.

**FIGURE 1:**
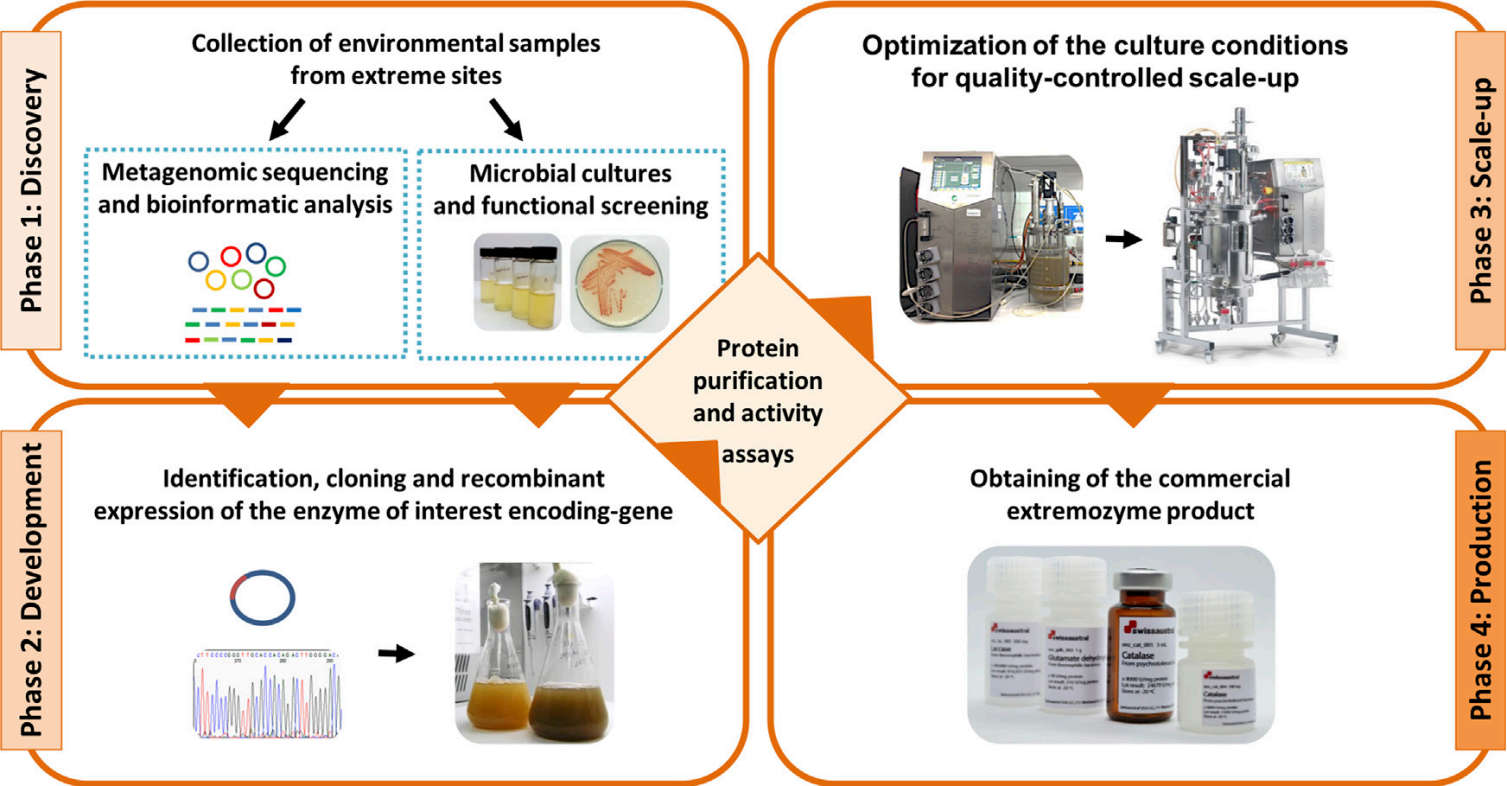
Strategy for the screening and production of new extreme biocatalysts from environmental samples. Culture dependent and independent approaches to discover novel extremozymes are depicted in the discovery phase.

After finding novel industrially relevant extremozymes by any of these two approaches, it is necessary to produce them in high amounts to obtain a commercial enzyme product that could be useful for biotechnological applications. Therefore, it is required to develop the recombinant version of the native enzyme of interest by cloning and expressing its coding gene in a suitable heterologous host-vector system, which allows to obtain higher productivity in shorter periods of time. Then, the recombinant extremophilic enzyme functionally overexpressed needs to be biochemically characterized. Finally, it is necessary to perform growth optimization for quality-controlled scale-up and downstream processing (Figure 1).

Here we describe a stepwise strategy based on the culture dependent functional approach, for the discovery, development, scale-up and production of three novel industrially relevant extremozyme products for the Research Market: a catalase, a laccase and an amine transaminase. The experimental work was carried out at Fundacion Biociencia, a Chilean non-profit scientific research institution, with over 20 years of experience dedicated to the study of extremophilic microorganisms and their bio-compounds.

## Methods

### Phase 1: Discovery of Extremophiles with Industrially Relevant Enzyme Activities

Environmental samples collected during different Scientific Expeditions to extreme sites are selected according to physicochemical characteristics required for a specific industrial process. Considering the nature of the samples and their original conditions, they are inoculated in different culture media applying specific selection pressures to enrich the culture with microorganisms with the desired characteristics. To this end, because of the growing demand for highly active catalases, microorganisms naturally exposed to environmental stresses that trigger the generation of reactive oxygen species (i.e., low temperatures, high UV radiation) were selected and functionally screened, since they must have an important antioxidant defense mechanism (such as an active catalase) to deal with those extreme conditions. Similarly, because thermostable and thermoactive laccases and amine-transaminases are currently largely required by several industries, thermophilic microorganisms from different extreme environments were selected and functionally screened for these enzyme activities.

- Psychrotolerant catalase producing microorganisms were screened in environmental samples collected from Elephant Island, Antarctica. Samples were cultivated at 8°C and pH 6.5 for up to 2 weeks. Then, cultures were exposed to UV-C radiation in a specially designed dark chamber for 2 h, to enrich microorganisms with better antioxidant defense mechanisms (Monsalves et al., 2020).
- Thermoalkaliphilic laccase producing microorganisms were searched in environmental samples collected from a geothermal site and cultivated at 50°C, pH 8.0 in media supplemented with lignin as an inductor of laccase activity. Then, functional screening was performed using agar plates containing 0.5 mM guaiacol for easy identification of laccase positive brown colored colonies (Sheikhi et al., 2012).
- Thermophilic amine-transaminase producing microorganisms were screened in environmental samples collected from fumaroles located in Whalers Bay, Deception Island, Antarctica. Samples were cultivated at 50°C and pH 7.6 for 24 h in media supplemented with 10 mM *α*-methylbenzylamine (MBA) as an inductor of amine-transaminase activity (Márquez and Blamey, 2019).

Extremophiles that were able to grow under the determined conditions were isolated through several rounds of serial dilutions to extinction, in conjunction with spread-plate techniques until a single morphotype was observed. Isolated strains were maintained by routinely sub-culturing in fresh growth medium and as 20% glycerol suspension stored at −80°C. Subsequently, a polyphasic approach was used to identify the isolated microorganisms (Ramasamy et al., 2014). In addition, whole genome sequencing of the most promising extremophiles was performed on Illumina Miseq platform using Nextera XT DNA libraries at Georgia Genomics Facility (Georgia, United States). The sequenced genomes were assembled, curated and annotated at Fundacion Biociencia. Then, identification of the catalase, laccase and amine-transaminase encoding genes was done through comprehensive bioinformatic analysis of the genome sequence of each selected extremophilic microorganism.

### Phase 2: Development of the Recombinant Versions of Native Extremozymes

Cloning, overexpression and downstream processing is somehow different for every protein. As general considerations, when aiming to develop an enzyme product, selecting an unpatented expression vector/host is relevant in order to prevent infringement of intellectual property. Similarly, the utilization of affinity tags for easier purification, or fusion partners for increased solubility, is not recommended due to their use is often licensed for commercial purposes. Furthermore, selection of the cell location for heterologous expression, needs to consider the culture volumen and the laboratory equipment available, either for cell disruption and fractionation, in the case of intracellular overexpression, or for supernatant concentration when extracellular. For recombinant expression in the well-known, mesophilic heterologous host *Escherichia coli*, catalase and laccase encoding genes were PCR-amplified from their native microorganism’s genomic DNA using specific oligonucleotide primers, while amine-transaminase was codon optimized and synthesized by Atum (California, United States) (Márquez and Blamey, 2019; Espina et al., 2021b). Then, they were cloned into expression vectors characterized for carrying IPTG-inducible T5 promoter and kanamycin antibiotic resistance gene for selection, with neither affinity tags nor signal peptide for extracellular secretion.

*E. coli* competent cells were chemically transformed with each expression vector carrying the sequence verified genes. The selected transformants were grown aerobically at 37°C, with shaking at 180 rpm until OD600 = 0.6–0.8, in 50 ml LB medium supplemented with 30 μg/ml kanamycin, plus 2 mM CuSO_4_ in the case of laccase. At this point, the heterologous expression of the recombinant enzymes was induced by the addition of 0.1–0.5 mM IPTG and the cultures were further incubated at 30°C for 6–12 h. The cells were harvested by centrifugation at 9000 g for 15 min at 4°C and resuspended in 5 ml lysis buffer. Then, cell disruption was carried out by ten 15 s bursts of sonication (Branson Sonifier 450), and the cell lysate was centrifuged at 14000 g for 30 min at 4°C in order to obtain the soluble crude extract. Extremozyme’s overexpression was evaluated by SDS-PAGE (Gallagher and Sasse, 1998) and specific enzyme assays:

- Catalase activity was measured spectrophotometrically by monitoring the decrease in absorbance at 240 nm, due to the transformation of H_2_O_2_ to H_2_O and O_2_. The reaction mixture for the standard assay contained 10 mM H_2_O_2_ and 50 mM potassium phosphate pH 7.0, in a total volume of 3 ml. One unit of catalase activity was defined as the decomposition of 1 µmol of H_2_O_2_ per minute (Beers and Sizer, 1952).
- Laccase activity was assayed spectrophotometrically following the oxidation of syringaldazine substrate to tetramethoxy-azo-bis-methylene-quinone (II) at 530 nm. The reaction mixture for the standard assay contained 0.216 mM syringaldazine and 100 mM potassium phosphate buffer pH 6.0 in a total volume of 3 ml. One unit of laccase activity was defined as a change in absorbance at 530 nm of 0.001 per minute, under the assay conditions (Lante et al., 2000).
- Amine-transaminase activity toward (S)-*α*-MBA was measured spectrophotometrically following the acetophenone formation at 245 nm (*ε* = 12 mM^−1^ cm^−1^) The reaction mixture for the standard assay contained 1 mM (S)-*α* methylbenzylamine, 1 mM pyruvate and 10 μM PLP and 100 mM Tris-HCl buffer, pH 8.0 in a total volume of 1 ml. One unit of amine-transaminase activity was defined as the amount of enzyme that catalyzed the formation of 1 μmol acetophenone from (S)-*α*-MBA and pyruvate in 1 min (Schätzle et al., 2009).

Protein concentration was measured by Bradford method, using a commercially assay kit (Bio-Rad, California, United States).

The recombinant catalase, laccase and amine-transaminase overexpressed in functional, soluble form with no affinity tags were purified by anion exchange chromatography at room temperature on an ÄKTA Pure FPLC system (GE Healthcare). The soluble crude extracts were diluted 10-fold in loading buffer and loaded onto a pre-equilibrated 25 ml Q-sepharose XK 16/20 column. The column was washed with 10 column volumes (CV) of the same buffer, followed by a linear NaCl gradient (0–1 M) for protein elution.

Studies on the pH and temperature dependence were made for each purified recombinant protein in order to biochemically characterize them in terms of optimum pH, optimum temperature and thermostability.

### Phase 3: Optimization and Scale-up of the Biomass and Protein

After obtaining the recombinant extremozymes of interest overexpressed in functional and soluble form, it was necessary to optimize its production at laboratory scale in order to obtain better biomass and protein yields for downstream analyses. This optimization process was done in 250 ml flasks and also in a 5L Biostat B-plus bioreactor (Sartorius). The most common parameters to optimize in order to ensure satisfactory levels of biomass and protein overexpression during the production of recombinant extremozymes are: Culture media, pH, growth temperature, agitation rate, aeration, inoculum volume, optimum fermentation volume, culture time, antibiotic concentration, as well as inductor concentration, time, and temperature of induction (Steinberg and Bursztyn, 2010; Espina et al., 2020).

To determine the optimal parameters, statistical tools such as Analysis of Variance (ANOVA) were used to assess if there were significant differences between the studied factors. In addition, the complementary methodologies: Design of Experiments (DoE) (Mandenius and Brundin, 2008) and Response Surface Methodology (RSM) (Myers and Montgomery, 2016), were used to avoid experimental biases and reduce the number of experiments needed to achieve optimal conditions. DoE was used to obtain a matrix of experiments that allow varying more than one variable at the same time, and RSM allowed the analysis of the results obtained from the designed matrix, enabling the study of how the interactions between the input variables influence the output responses. Statgraphics Software (Virginia, United States) was used to determine the DoE and to analyze the surface of response using the biomass yield and enzyme activity in order to find the optimal conditions for each of the different enzymes. The combination of the parameters that allow to achieve the highest yield of biomass and recombinant enzyme were successfully determined for catalase and laccase, while for the amine-transaminase these experiments are currently ongoing. When using DoE and RSM methods, the obtained data are evaluated and analyzed to determine if there are significant differences between them and thus, validate if the method was successfully optimized or not. All analyses were carried out using a confidence interval of 95%, which allows to observe significant differences between biomass production, an indicator of a successful optimization.

After the process was optimized for catalase and laccase expression using bench scale reactors, the cultures were then scaled-up in order to replicate the behavior and results obtained in laboratory scale. Usually this increase corresponds to a volume ratio of 1/10 for a consecutive scaled-up (Votruba and Sobotka, 1992). Also, it is important to maintain the same conditions determined during the optimization and to use bioreactors that maintain geometrical similarity, and constant relations in their dimensions and lengths. The scale-up was considered to be completed when the yield of biomass and enzymes was similar in different scales and this was reproducible.

Then, the scaled-up cells were harvested by centrifugation at 9000 g, and disrupted using a high-pressure homogenizer (Constant Systems Cell Disruptor). The crude extract was obtained after ultracentrifugation at 30000 g for 20 min at 4°C. Due to their extremophilic origin an thermal stability, the intracellularly overexpressed catalase and laccase were purified using a heat shock process first, optimized in time and temperature using multifactorial ANOVA. This first purification step allows efficient separation from many native *E. coli* proteins that precipitate with increased temperatures. Then, anion exchange chromatography was used as a second step of purification following the protocol previously described, using a 450 ml Q-sepharose resin in XK 50/60 column. It is important to mention that the final purity required for a commercial extremozyme product is directly related to its target applications.

Stability studies of the purified recombinant proteins were made by assaying enzyme activity in time, at two different storage temperatures: 4°C and −20°C. These experiments were made for each protein in solution and also as lyophilized powder as this format could offer easier transportation and sometimes better stability than liquid format.

### Phase 4: Obtaining the Enzyme Product

Achieving reproducibility in each batch in relation to biomass and/or protein production is very important, as the whole process must be validated to ensure that it will consistently generate a product meeting its predetermined specifications. Hence, after successful scale-up, it was necessary to determine and assess quality control points (QCP) in order to obtain a final product with a reproducible quality (Nummer et al., 2015; Table 1). If the final product complies with the Quality Control Certificate parameters and Product Datasheet specifications, it is labeled and delivered with all the necessary paperwork (e.g., Analysis Certificate, Material Safety Data Sheet and Certificate of Origin) to a final user or an appropriate distributor for its commercialization (Espina et al., 2020).

**TABLE 1:** Quality control points (QCP) defined for enzyme production.

Quality control points	Step	Control point
QCP1	Seed culture preparation	Control of biomass growth from lab-scale bioreactor (wet biomass yield, g/L)
QCP2	Biomass production	Control of biomass growth from a larger bioreactor (wet biomass yield, g/L)
QCP3	Protein extract	Control of enzyme activity (U/ml) and protein concentration (mg/ml) of the extract prior purification
QCP4	Protein purification	Control of specific activity (U/mg) of the purified protein
QCP5	Final product processing	Control of specific activity (U/mg) and protein concentration (mg/ml) of the final product

### Comparison with other Commercial Enzymes

The lyophilized commercial product enz_cat_004 (Swissaustral) was compared with other commercial enzymes: catalase from *Aspergillus niger* (Sigma C3515) and catalase from bovine liver (Sigma C40). Each enzyme was assayed as described above, at different temperatures, between 20°C and 70°C, at their optimum pH reported by each manufacturer in their data sheets.

The lyophilized commercial product enz_lac_005 (Swissaustral) was compared with other commercial enzymes: Laccase from *Aspergillus sp.* (Sigma SAE0050), laccase from *Aspergillus oryzea* (Novozym 51003) and laccase from *Trametes versicolor* (Sigma 38429). Each enzyme was assayed at different temperatures, between 20°C and 80°C, at their optimum pH reported by each manufacturer in their data sheets.

## Results

### Catalase

Catalases (EC 1.11.1.6) are enzymes that act on hydrogen peroxide as an acceptor, characterized by utilizing a second H_2_O_2_ molecule as the electron donor, to catalyze its dismutation into H_2_O and O_2_ without consuming cellular reducing equivalents (Heck et al., 2010). These enzymes have various biotechnological applications as hydrogen peroxide is extensively used as a powerful oxidizing, bleaching, or sterilizing agent by many industries, including food, textile, pulp and paper, biomedicine, clinical diagnostic, bioremediation (Lončar and Fraaije, 2015; Espina et al., 2021a).

After functional screening, a psychrotolerant and radiation resistant bacterium was isolated from environmental samples collected from Elephant Island, Antarctica. This microorganism was identified as *Serratia* sp. I1P, with optimal growth conditions at 22°C, pH 7.0–9.0, 2–6% NaCl (Monsalves et al., 2020)*.* A novel catalase was purified from the crude extract and biochemically characterized. The native enzyme was active in a wide range of temperatures (20–70°C), showing optimal activity at 50°C and pH 7.0, with a half-life of 7 h when incubated at 50°C, which is remarkable considering its psychrotolerant origin (Monsalves et al., 2020).

Optimization of the recombinant overexpression of this enzyme showed that the best growth conditions were REC-P culture medium, at 23°C, pH 7.0, 15% pO_2_ concentration and 16 h cultivation. The optimal culture medium and physico-chemical conditions were determined during the optimization phase done in 5L Biostat B-plus bioreactor, and then scale-up was done in pilot scale using a 40L Biostat C-plus bioreactor (Sartorius).

A comparison of the results of biomass yield, enzymatic yield, and specific activity obtained during each step of the catalase product development is presented in Table 2. The optimized and scale-up recombinant expression allow to raise up the values of activity over 6 times, biomass production increase fivefold and enzyme production over 50 times.

**TABLE 2:** Comparison of native and recombinant enzyme production.

	*Serratia* sp. I1P catalase	*Bacillus* sp. FNT laccase	*Albidovulum* sp. SLM16 amine-transaminase
**Biomass Yield**
Native biomass (5L Biostat-B Bioreactor)	5 g/L	10 g/L spore, 16 g/L biomass	8 g/L
Recombinant biomass (5L Biostat-B Bioreactor)	5 g/L	20 g/L	30 g/L
Optimized recombinant biomass (5L Biostat-B Bioreactor)	16 g/L	25 g/L	Ongoing experiments
Scaled-up recombinant biomass (40L Biostat-C Bioreactor)	25 g/L	25 g/L	Ongoing experiments
**Enzymatic Yield**
Native enzymatic yield (5L Biostat-B Bioreactor)	8.5 mg protein/L culture	4.4 mg protein/L culture	12 mg protein/L culture
Recombinant enzymatic yield (5L Biostat-B Bioreactor)	100 mg protein/L culture	200 mg protein/L culture	28 mg protein/L culture
Optimized recombinant enzymatic Yield (5L Biostat-B Bioreactor)	550 mg protein L culture	500 mg protein/L culture	Ongoing experiments
Scaled-up recombinant enzymatic Yield (40L Biostat-C Bioreactor)	443.7 mg protein/L culture	450 mg protein/L culture	Ongoing experiments
**Specific Activity**
Native specific activity (5L Biostat-B Bioreactor)	1301 U/mg	1000 U/mg	0.52 U/mg
Recombinant specific activity (5L Biostat-B Bioreactor)	4000 U/mg	200000 U/mg	6 U/mg
Optimized specific activity (5L Biostat-B Bioreactor)	10000 U/mg	400000 U/mg	Ongoing experiments
Scaled-up specific activity (40L Biostat-C Bioreactor)	≥8600 U/mg	≥300000 U/mg	Ongoing experiments

The quality control system was applied in every step of the process, which allows to obtain a final enzymatic product with constant reproducibility. Furthermore, stability tests performed showed that at 4°C, the liquid enzyme product maintained more than 60% of its activity after 260 days and more than 80% of its activity after 320 days when stored at −20°C. The lyophilized enzyme kept the reported activity (>8000 U/mg) at least 2 years, stored at −20°C.

This novel thermostable catalase from psychrotolerant origin is currently commercially available as an enzyme product from Swissaustral LLC. Currently, it is available in two formats: liquid (enz_cat_001) and lyophilized (enz_cat_004) with ≥65000 U/ml and ≥8000 U/mg, respectively.

**FIGURE 2:**
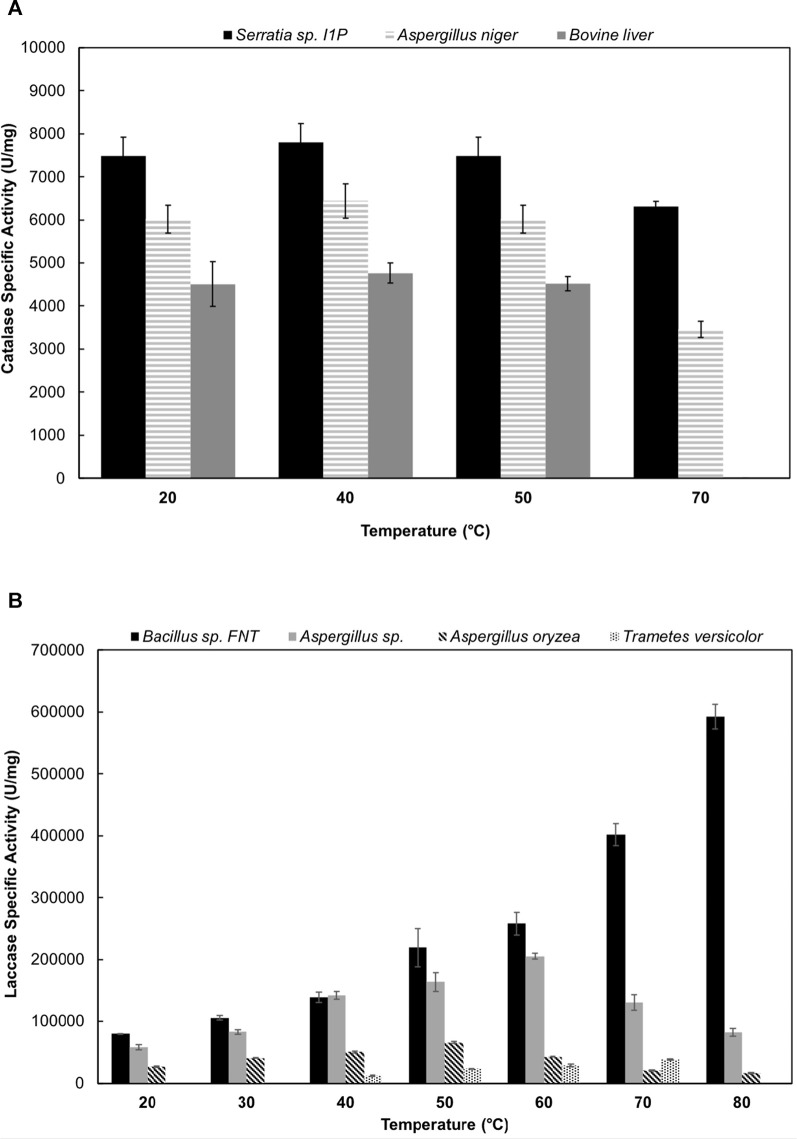
Comparison of commercial catalases **(A)** and laccases **(B)** at different temperatures. Each commercial enzyme was assayed at their optimum pH, as reported by each manufacturer in their data sheets. The enzyme assays used were described in the method section for each enzyme. One unit (U) of CAT activity was defined as the decomposition of 1 µmol of H_2_O_2_ per minute. One unit (U) of laccase activity was defined as a change in absorbance at 530 nm of 0.001 per minute, under the assay conditions. Error bars indicate standard deviation of triplicate measurements.

The comparison of the lyophilized product (enz_cat_004) with two other commercial catalases from fungal and bovine origin (Figure 2A), proved that the catalase enzyme, discovered, and developed at Fundacion Biociencia is highly active in a broad range of temperatures (20°C—70°C). This novel extremozyme product is a promising candidate for potential biotechnological applications, as it is better suited than mesophilic enzymes to resist the harsh conditions found in many industrial processes. Unfortunately, commercial catalases as most enzymatic products available on the market, do not report information regarding their purity that could allow further comparisons between products. The catalase product from Swissaustral is currently commercialized at a purity of at least 53% (Supplementary Table S1) for biotechnological applications. Additionally, its purity level could be increased up to 95% if required for a specific application, such as biomedical and clinical diagnostic.

### Laccase

Laccases (EC 1.10.3.2), are enzymes that catalyze the oxidation of a wide array of phenolic and non-phenolic compounds, only requiring oxygen as co-substrate and releasing water as the sole by-product (Riva, 2006). Due to their broad substrate range, versatility and ease of use, laccases have great biotechnological potential and are useful in many applications such as: Food industry (Osma et al., 2010), pulp and paper (Singh and Arya, 2019), organic synthesis (Mogharabi and Faramarzi, 2014), bioremediation (Strong and Claus, 2011), biofuels (Kudanga and Le Roes-Hill, 2014), textile industry (Rodríguez Couto and Toca Herrera, 2006), biomedical and pharmaceuticals (Mohit et al., 2020), and biosensors (Castrovilli et al., 2019).

After functional screening, a thermoalkaliphilic spore-forming bacterium was isolated from an environmental sample obtained from a hot spring in a geothermal site. This microorganism was identified as *Bacillus* sp. FNT, with optimal growth conditions at 50°C, pH 8.0. A novel laccase was found in the spores, and it was successfully purified and biochemically characterized. The recombinant version of this enzyme exhibits remarkably high specific activity at 70°C, pH 6.0, and is active in a wide range of temperature (20–90°C) with a half-life of 3 h when incubated at 60°C (Espina et al., 2021a).

Optimization of the recombinant overexpression, showed that the best growth conditions were TBA culture medium supplemented with 2 mM CuSO_4_, at 30°C, pH 7.0, 15% of pO_2_ concentration and 23 h cultivation. The optimal culture medium and physico-chemical conditions were determined during the optimization phase done in 5L Biostat B-plus bioreactor, and then scale-up was done in pilot scale using a 40L Biostat C-plus bioreactor.

A comparison of the results of biomass yield, enzymatic yield and specific activity obtained during each step of the laccase product development is presented in Table 2. The optimized and scale-up recombinant expression raise up the values of activity over 300 times, biomass production increase 2.5 times and enzyme production over hundredfold.

The quality control system was applied in every step of the process, which allows to obtain a final enzymatic product with constant reproducibility. Furthermore, stability tests performed showed that the lyophilized enzyme product maintained the same reported activity at least for 11 months at −20°C. This novel thermostable laccase from thermoalkaliphilic origin is currently commercially available as an enzyme product from Swissaustral LLC. At the moment, it is available only in lyophilized format (enz_lac_005) with ≥300000 U/mg. Stability tests of liquid format are currently under evaluation.

The comparison of the product (enz_lac_005) with three other commercial laccases from fungal origin available in the market (Figure 2B), proved that the laccase enzyme discovered and developed at Fundacion Biociencia is highly active in a broad range of temperatures, confirming that this novel extremozyme product is a promising candidate for potential biotechnological applications. Swissaustral laccase is currently commercialized at a purity of at least 78% (Supplementary Table S1).

### Amine-transaminase

Transaminases (EC 2.6.1. X), are a large pyridoxal 5’-phosphate dependent group of enzymes capable of catalyzing the stereoselective reversible transfer of an amino group from a donor substrate to the carbonyl carbon atom of an acceptor substrate (Ferrandi and Monti, 2018). Amine-transaminases (ATA) are a subgroup of *ω*-transaminases enzymes with the ability to perform reductive amination on prochiral ketones using a wide range of primary amine donors and acceptors. Consequently, these enzymes are of great industrial interest especially for the pharmaceutical, fine chemicals and agrochemical industries, since they are a promising alternative to replace the enantioselective chemical synthesis of optically pure chiral amines, which is currently done using transition metals as catalysts (Ferrandi and Monti, 2018; Kelly et al., 2020).

After functional screening of transaminase activity, a thermophilic bacterium was isolated from an environmental sample collected from coastal fumaroles emerging at the shore at Whalers Bay in Deception Island, Antarctica. This microorganism was identified as *Albidovulum* sp. SLM16, with optimal growth conditions at 55°C, pH 6.5–8.0, 1–3% NaCl (Márquez and Blamey, 2019). A novel (S)-ATA enzyme was purified from the crude extract and biochemically characterized. The recombinant version of this enzyme has been obtained with a purity of 99.7% (Supplementary Table S1), and found to be active in a wide range of temperatures (20–70°C), showing optimal activity at 65°C and pH 9.5, with a remarkable thermostability maintaining 80% of its specific activity after 5 days of incubation at 50°C (Marquez and Blamey, 2019).

The optimal culture medium for this enzyme overexpression was determined to be TBA. Further optimization of the growth conditions for recombinant overexpression of this enzyme is currently ongoing for subsequent scale-up.

A comparison of the results of biomass yield, enzymatic yield and specific activity obtained during the ATA development is presented in Table 2. The non-optimized recombinant expression raises up the values of activity over 11 times, biomass production increases over 3.5 times and doubles the enzyme production. These are very promising results indicating that there is still room for improvement after optimization leading to scale-up of enzyme production. The quality control system has to be applied in every step of the process in order to obtain the final enzymatic product with constant reproducibility. Then, stability tests at different storage temperatures in time, and comparison with another commercial ATA will also be performed before obtaining the final enzyme product.

To date, advances are being consistently made in order to develop this industrially relevant, thermophilic and highly thermostable ATA, as a commercial extremozyme product in the near future.

## Conclusion

In the increasing need to replace toxic chemical catalysis for biocatalysis, enzymes derived from microorganisms isolated from extreme environments have called the attention. Their remarkable properties allow them to carry out reactions under nonstandard conditions, being highly suitable for industry. Consequently, the search and discovery of novel enzymes will drive future biotechnological innovation improving current industrial processes.

Even though culture independent bioprospection based on metagenomic sequencing and analysis might help to obtain novel enzyme products in a faster way, many times when interesting encoding genes are selected directly from the genome sequence, there is a risk that they would not necessarily display the desired activity when heterologously overexpressed. Undoubtedly, one of the main advantages of the classic functional approach is that it ensures that there is actually an enzyme with the desired characteristics being functionally expressed in the native microorganism. This increases the probability of producing a recombinant version of the enzyme that keeps similar characteristics to the native one. As presented in this brief research report, using a stepwise strategy based on a culture dependent functional approach, has successfully led to the development of interesting extremozyme commercial products from environmental samples.

## Data Availability

The raw data supporting the conclusion of this article will be made available by the authors, without undue reservation.
