# Supplementary material for: From the Discovery of Extremozymes to an Enzymatic Product: Roadmap Based on Their Applications
Source: Front Bioeng Biotechnol. 2022 Jan 12;9:752281. doi: 10.3389/fbioe.2021.752281 (PMC8790482; doi:10.3389/fbioe.2021.752281)
Supplement: Supplementary file 1 [file Table1.DOCX]

**Supplementary Material**

From the discovery of extremozymes to an enzymatic product: Roadmap based on their applications

**Giannina Espina ^1^, Sebastián A. Muñoz-Ibacache^1^, Paulina Cáceres-Moreno^1^, Maximiliano J. Amenabar^1^, Jenny M. Blamey^*1,2^**

^1^ Fundación Biociencia, José Domingo Cañas 2280, Ñuñoa, Santiago, Chile

^2^ Facultad de Química y Biología, Universidad de Santiago de Chile, Alameda 3363, Estación Central, Santiago, Chile.

*** Correspondence:**Corresponding Author: [jblamey@bioscience.cl](mailto:jblamey@bioscience.cl), gespina@bioscience.cl

**Table S1.** **Purity level of native and recombinant enzymes.** These values were estimated from SDS-PAGE images using ImageJ software.

| **Enzyme** | **Purity (%)** |
| --- | --- |
| Native catalase | 45.7 |
| Recombinant catalase | 53.5 |
| Native laccase | 72.8 |
| Recombinant laccase | 78.1 |
| Native amine-transaminase | 42.3 |
| Recombinant amine-transaminase | 99.7 |

The percentage of purity were estimated by densitometry of the protein bands observed in SDS-PAGE using ImageJ software.
